# Effects of Palm Kernel Cake on Nutrient Utilization and Performance in Confined Cattle, Sheep and Goats: A Comparative Meta-Analytical Approach

**DOI:** 10.3390/ani15182764

**Published:** 2025-09-22

**Authors:** Julián Andrés Castillo Vargas, Anaiane Pereira Souza

**Affiliations:** 1 Center for Agrarian and Biological Sciences, State University of the Vale of Acarau, Acaraú 62580-000, CE, Brazil; 2 Institute of Studies of the Humid Tropic, Federal University of the South and Southeast of Para, Xinguara 68555-251, PA, Brazil; anaianesouza@unifesspa.edu.br

**Keywords:** by-product, confinement, digestibility, intake, quantitative approach, ruminant, species

## Abstract

This meta-analysis explored the relationship of palm kernel cake inclusion level (PKC_Inclusion_) with nutrient utilization and performance in cattle, goats and sheep under confinement. For this purpose, a dataset with 51 studies was constructed by using the PRISMA (Preferred Reporting Items for Systematic Review and Meta-Analysis) Protocol. Relationships of PKC_Inclusion_ with dry matter (DM), crude protein (CP), EE (ether extract), NDF (neutral detergent fiber) and TDN (total digestible nutrients) intake and digestibility, as well as with ADG (average daily gain) and FE (feed efficiency), were explored under a mixed model approach, considering the species and study as fixed and random effects, respectively. Data revealed independent (*p* < 0.075) relationships of EE and TDN intake and digestibility with PKC_Inclusion_ for cattle, goats and sheep. However, the relationship of CP intake and digestibility with PKC_Inclusion_ did not differ (*p* > 0.114) between ruminant species. Goats and sheep demonstrated similar quantitative patterns for DM and NDF intake but different quantitative patterns (*p* < 0.037) from those observed for cattle with the increase in PKC_Inclusion_ in the diet. Regarding performance, FE responses were similar between cattle and sheep but differed (*p* < 0.001) from those observed for goats; however, ADG demonstrated similar (*p* = 0.243) decreasing rates among ruminant species in function of dietary PKC_Inclusion_. In conclusion, PKC_Inclusion_ has differential effects on the intake and digestibility of DM and most of the nutritional components in confined cattle, goats and sheep, except for CP. The data reported herein could be used in future nutritional models to allow for the better use of alternative feedstuffs, such as PKC in productive ruminants under confinement.

## Simple Summary

Palm kernel cake (PKC) is a by-product derived from plant oil extraction with nutritional and performance responses reported in different ruminant species under confinement. However, the literature suggests contradictory results, even within the same ruminant species. Hence, this study conducted a meta-analysis to explore if dietary PKC inclusion has differential effects on dry matter (DM) intake and nutrient utilization, as well as in performance, across confined cattle, goats and sheep. Data revealed that the same dietary PKC inclusion level may result in different responses of DM and most nutrients intake and digestibility across explored ruminant species, except for crude protein utilization. Performance results suggest that these ruminant species may have similar average daily gain decreasing rates but differences in feed efficiencies at similar PKC inclusion levels. Elucidating the effects of PKC inclusion on nutrient utilization and performance across confined ruminants may help optimize animal production profitability and enhance the palm oil extraction production chain from a sustainable standpoint.

## 1. Introduction

The use of non-conventional feedstuffs in ruminant production has increased in the last years, reducing production costs as well as the competition of animal feed chain production with the human food chain production [1]. Particularly, by-products derived from the biodiesel industry have gained special attention, considering their worldwide expansion and technification [2]. Among by-products derived from the biodiesel industry, palm kernel cake (PKC; *Elaeis guineensis* Jacq.) represents a suitable feedstuff for animal production, as its nutrient composition is compatible with the digestive physiology of the ruminant [3]. Under that scenario, PKC may be potentially used in ruminant diets, particularly with promissory results in confined animals [4]. As a result, different studies evaluating its effects on the nutrient utilization and performance in ruminant species (e.g., cattle, sheep and goats) under confinement have been conducted in the last years [5,6,7].

In cattle, diverse studies suggest that PKC can reduce the DM intake and digestibility in a dose-dependent manner [4,8,9]. However, dietary PKC_Inclusion_ could be a promissory strategy for increasing the feed efficiency ratio, without detrimental effects on the gain and final weight [4,8]. In small ruminants (i.e., sheep and goats), it is suggested that the dietary inclusion of PKC may have positive, negative or no effects on intake, digestibility and performance. In goats, it has been reported that PKC does not affect DM intake and nutrient digestibility [7], with concomitant positive effects on weight gain and feed efficiency [10]. In sheep, several studies have revealed that PKC_Inclusion_ in the diet may have detrimental effects on the nutrient utilization and performance [5,11]. Thus, based on the aforementioned facts, it can be suggested that the dietary effects of PKC may differ between confined ruminant species.

It has already been demonstrated in early studies that ruminant species have differences in ingestive behavior and digestive anatomy [12,13,14]. Additionally, cattle, sheep and goats differ in their digestion patterns for fiber and fat [15,16] and they also have distinct nutrient requirements [17,18]. These differences could be critical when including PKC in the diet of ruminants due to it being a high lipid (~190 g/kg DM) and neutral detergent fiber (NDF; ~717 g/kg DM) source [19]. Also, PKC has high levels of Cu (~30 ppm), which could be harmful for ruminants, particularly for sheep [20]. Hence, understanding the differential impacts of dietary PKC_Inclusion_ among cattle, sheep and goats could provide valuable insights regarding the efficient use of this feedstuff in their diets. Nevertheless, until now, there are no studies exploring those differences from a quantitative and robust approach.

The exploration of the effects of species on nutrient utilization and performance in ruminants could be successfully evaluated by using a meta-analytical approach. This statistical technique allows for evaluating specific fixed effects in datasets constructed from different studies while isolating intra and inter-study random effects [21,22]. Hence, the objective of this study was to evaluate the effects of species on the relationship of dietary PKC_Inclusion_ with dry matter (DM) and nutrient utilization and performance in confined cattle, sheep and goats. We hypothesize that dietary PKC_Inclusion_ has different impacts on DM and nutrient utilization and performance in the aforementioned ruminant species under confinement.

## 2. Materials and Methods

### 2.1. Literature Search

A dataset was developed to explore the effects of species on the relationship between dietary PKC_Inclusion_ and nutrient utilization and performance in confined ruminants. For this purpose, documents (e.g., articles, theses and dissertations) were searched in online scientific platforms (Google Scholar, Science Direct, Scopus, PubMed Central and Web of Science). Selection was expanded to documents different from already published articles, because the probability of publication bias is reduced when restrictions are reduced during the literature search [23,24]. The terms used for selecting the studies were “cattle”, “bovine”, “sheep”, “ovine”, “lamb”, “goat”, “caprine”, “palm kernel cake”, “nutrient”, “intake”, “consumption”, “confined”, “confinement”, “ingestion”, “nutrient utilization”, “digestibility”, “average daily gain”, “feed efficiency” and “performance”. In the initial phase of study searching, 182 scientific documents were obtained and transferred to a database in the Mendeley software (Mendeley Desktop 1.19.8) for a filtering process.

### 2.2. Selection of Studies and Development of the Dataset

**Figure 1:**
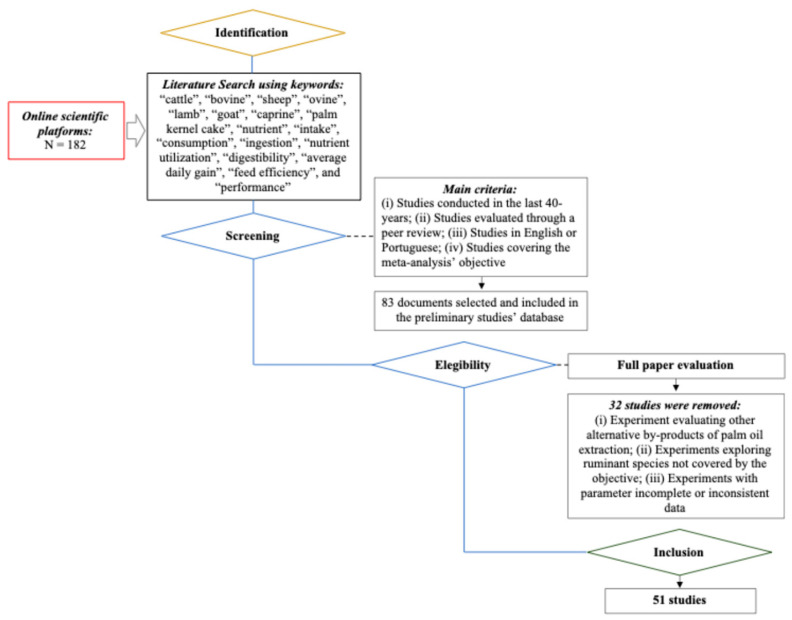
Flowchart of the literature selection process in accordance with the PRISMA (Preferred Reporting Items for Systematic Review and Meta-Analysis) Protocol.

A selection within the collected studies was developed for ensuring data integrity and that each study was in accordance with the objectives of meta-analysis. In a preliminary screening, duplicate papers or those with titles out of the meta-analysis’ scope were removed from the database. From that procedure, 83 studies were considered suitable and retained in the Mendeley software for further selection. The PRISMA (Preferred Reporting Items for Systematic Review and Meta-Analysis) Protocol [25] was used for selecting the studies with data to be extracted (Figure 1). The studies’ selection was based on the following eligibility criteria: (i) studies within the last forty years, (ii) studies evaluated through a peer-review process (including journal articles, master’s theses and doctoral dissertations), (iii) studies written in English or Portuguese, with an English-language abstract and (iv) studies aiming to evaluate the effects of PKC_Inclusion_ on nutrient utilization and/or the performance in confined cattle, goats or sheep.

The dataset was built based on 51 studies. The list of references and details of the studies (Table S1) included in the meta-analysis can be found in Supplementary Materials and the descriptive statistics of quantitative variables explored can be found in Table 1.

This dataset comprised variables of study characteristics (i.e., study’s number, bibliographic data, number of replications per study, study’s objective, species (i.e., cattle, goats, and sheep), and mean BW (mBW; kg; calculated as a mean of initial and final BW)) and quantitative variables related to the nutritional relationship between the level of PKC_Inclusion_ (g/kg DM) and daily dry matter (DM) intake (DM_Intake_; g/kg BW^0.75^), crude protein (CP) intake (CP_Intake_; g/kg BW^0.75^), fat (expressed as ether extract (EE), intake (EE_Intake_; g/kg BW^0.75^), neutral detergent fiber (NDF) intake (NDF_Intake_; g/kg BW^0.75^), total digestible nutrients (TDN) intake (TDN_Intake_; g/kg BW^0.75^), DM digestibility (DM_Dig_; g/kg DM), CP digestibility (CP_Dig_; g/kg DM), EE digestibility (EE_Dig_; g/kg DM), NDF digestibility (NDF_Dig_; g/kg DM), TDN concentration (TDN_Conc_; g/kg DM), average daily gain (ADG; g/day), and feed efficiency (FE; g gain/kg feed).

Some variables in the dataset were reported in different units across the studies. Therefore, when necessary, conversions’ unit operations were used to standardize the units of variables in the dataset. For example, when a DM or nutrient intake value was reported in g/day, it was converted to g/kg BW^0.75^ by dividing the value by the mean BW raised to the power of 0.75 (Metabolic weight; BW^0.75^). In addition, when DM or nutrient digestibility was reported as a percentage (%), it was converted to g/kg DM by multiplying the value by 10. All inclusion levels of PKC were calculated and expressed as the PKC_Inclusion_ in the total diet, independent of the dietary PKC inclusion form (e.g., in the supplement or total mixed ration (TMR)). Also, DM and nutrient intake were expressed considering metabolic body size (i.e., BW^0.75^) as a basis. Traditionally, this unit has been used for comparing feed intake among productive ruminant species [26].

When a study did not provide all the quantitative data required for the standardization of the units, the data register was not included in the meta-analysis. Considering that all selected studies did not explore the effects of PKC_Inclusion_ on nutritional and performance responses across the ruminant species, the number of data points can vary across the regression relationships explored. The chemical composition of experimental diets can be assessed in Supplementary Files (Table S2).

### 2.3. Statistical Analysis

All statistical procedures were conducted using SAS software (SAS Institute Inc., Cary, NC, USA; version 9.4). The PROC MEANS was used for performing an initial descriptive data analysis. Thereafter, multiple quantitative mathematical relations of the PKC_Inclusion_ level with nutrient utilization and performance variables were explored under a mixed model approach using the PROC MIXED. Heterogeneity among the studies and publication bias were evaluated using the Higgins’ and Thompson’s (*I*^2^) statistic and the Egger test, respectively, implementing the meta package of R (Version: 8.2-0) [27].

In the analyses conducted, the study was considered a random effect [28], while the species was considered a fixed effect. At this time, it is important to point out that gender was tested in the model as a random effect, but it had no significant influence on the relationships explored (*p* > 0.10). Hence, it was removed from the models for parsimony. Thus, the general equation used was

Y_ijk_ = B_0_ + B_1_X_ijk_ + B_2_X_2ijk_ + s_i_ + b_i_X_ijk_ + e_ijk_,

where Y_ijk_ is the expected response for a dependent variable, B_0_ is the overall intercept across all studies (fixed effect), B_1_ is the linear regression coefficient (fixed effect), B_2_ is the quadratic regression coefficient (fixed effect), X_ijk_ is the independent variable, s_i_ is the study effect (random effect), bi is the random effect of the study on the regression coefficients of Y_ijk_ on X_ijk_ and e_ijk_ is the residual error. The quadratic term was removed from the model when non-significant (*p* > 0.10) [29].

The restricted maximum likelihood (REML) estimation method was used for conducting the mixed regression analysis. When the fixed effect (species) was significant (*p* ≤ 0.10), that is, when there was an influence of the fixed effect on at least one significant parameter in the model, independent equations were produced for each species or pair of species. Otherwise (i.e., when the fixed effect did not influence at least one parameter in the model), one equation was produced for three species.

The estimation of linear and quadratic regression coefficients for X_ijk_ was conducted using the ESTIMATE statement, and pairwise comparisons between species were conducted using the CONTRAST statement of SAS. The normal distribution of the errors for all variables included in the quantitative relationships and variance homogeneity (i.e., residual errors are additive in the native scale; [30,31]) was checked based on the graph of studentized residuals for response variables by using the standard SAS output [28]. Outliers and influence values were detected and deleted when their normalized residuals were >|3| and their Cook’s distances were >0.1, respectively. Considering that variance across studies is unequal, studies were weighted by the number of experimental units [32,33] using the WEIGHT statement of SAS [34].

The corrected Akaike’s information criterion (AIC_c_) was obtained from the SAS output and was used for selecting the most reliable mathematical relationships, in which equations with the best goodness-of-fit were represented by the lowest AIC_c_. The Model Evaluation System (MES v.3.2.2, http://nutritionmodels.com/mes.html, accessed on 3 June 2025) was used to compare predicted and observed values of the models, thus evaluating their adequacy (e.g., accuracy and precision). Adequacy was assessed by calculating the concordance correlation coefficient (CCC), the coefficient of determination (R^2^) and the root mean square error (RMSE) [35]. Models with high, moderate and low precision were assumed when R^2^ ≥ 0.50, 0.50 > R^2^ ≥ 0.30 and R^2^ < 0.30, respectively. In addition, models with a CCC near to 1 and smaller RMSE values were considered accurate.

## 3. Results

**Figure 2:**
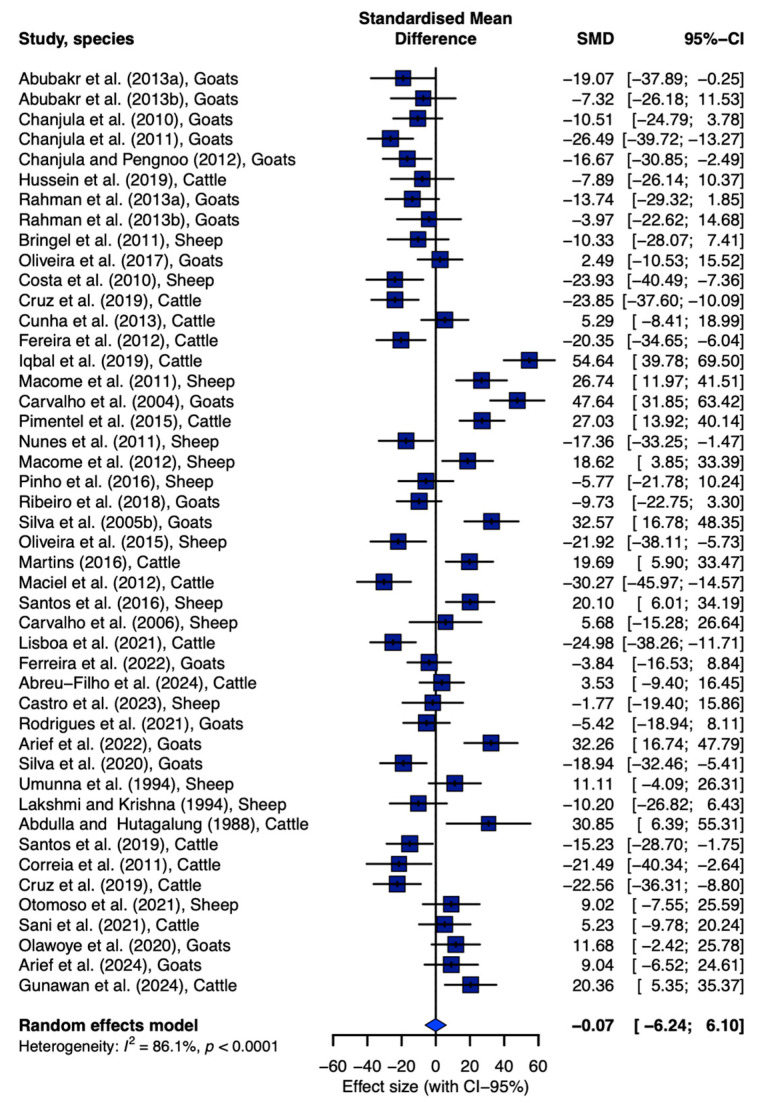
Forest plot of the effects of PKC on dry matter intake (DMIntake; g/kg BW^0.75^) in cattle, goats and sheep. The blue diamond represents the general random effect across studies. Studies with a 95% confidence limit crossing the zero point indicate statistical difference between the estimation of individual (study) and overall random effects.

Fifty-one studies were selected for the current meta-analysis, involving 1268 replications: 39% of studies were conducted with cattle, 35% with goats and 26% with sheep. Most of the studies with cattle (75%) and goats (94%), as well as all studies included in the dataset with sheep (100%), reported DM_Intake_. Hence, this was the main reason for selecting DM_Intake_ to evaluate the potential existence of heterogeneity across studies and publication bias. Figure 2 shows the forest plot for the meta-analysis involving the data of the DM_Intake_ of cattle, goats and sheep submitted to increasing levels of PKC. The I^2^ statistics appointed high (86.1%) and significant (*p* < 0.01) heterogeneity across studies, justifying the use of a random-effects model, in which study was considered as a random effect. Additionally, Egger’s test for statistical asymmetry revealed the absence of publication bias (*p* = 0.711).

### 3.1. Dry Matter and Nutrients Intake

**Figure 3:**
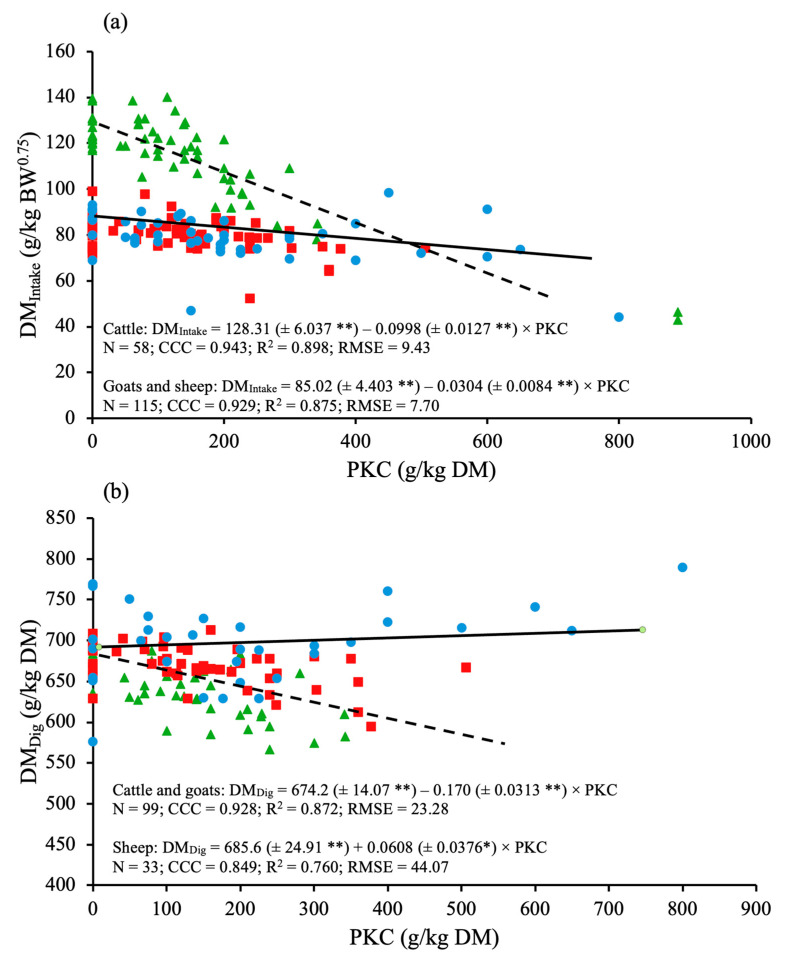
Effects of palm kernel cake inclusion on (**a**) dry matter intake (DMIntake; g/kg BW^0.75^) and (**b**) dry matter digestibility (DMDig; g/kg DM) in cattle (▲), goats (■) and sheep (●). N = total number of treatment means used for fitting the equation after data depuration. Values within parentheses correspond to the standard error of the parameters in the equation. ** *p* < 0.05 and * 0.05 ≤ *p* < 0.10. CCC = concordance correlation coefficient. R^2^ = coefficient of determination. RMSE = root mean square error. *p* values (effect of species): Intercept: *p* < 0.001, Slope (linear): *p* < 0.001.

Mixed regression analysis revealed that there was a linear relationship between DM_Intake_ and PKC_Inclusion_, in which the intercept and slope were influenced (*p* < 0.001) by the species (Figure 3a). However, the slope (*p* = 0.534) and the intercept (*p* = 0.973) of the aforementioned relationship were not different between goats and sheep. Therefore, one independent equation for cattle and another for goats and sheep were built. In cattle, goats and sheep, DM_Intake_ (g/kg BW^0.75^) decreased linearly (*p* < 0.001) as PKC_Inclusion_ (g/kg DM) increased (Figure 3a). There was a linear relationship between CP_Intake_ and PKC_Inclusion_ without the influence (*p* = 0.807) of species (Table 2). Therefore, one linear equation for cattle, goats and sheep was produced. In the aforementioned ruminant species, CP_Intake_ (g/kg BW^0.75^) decreased linearly (*p* < 0.001) as PKC_Inclusion_ (g/kg DM) increased (Table 2).

Data revealed that species affected (*p* = 0.075) the relationship between EE_Intake_ and PKC_Inclusion_, resulting in a quadratic pattern of the EE_Intake_ for cattle, whereas for sheep, it exhibited a linear pattern (Table 2). Hence, independent equations for cattle and sheep were produced (Table 2). In cattle, EE_Intake_ (g/kg BW^0.75^) showed a quadratic increasing pattern (*p* < 0.001) with a maximum at 199.3 g PKC/kg DM (Table 2). In sheep, EE_Intake_ (g/kg BW^0.75^) increased linearly (*p* < 0.001) as PKC_Inclusion_ (g/kg DM) increased (Table 2). Regarding goats, there was not a significant effect (*p* = 0.140) of PKC_Inclusion_ (g/kg DM) on EE_Intake_ (g/kg BW^0.75^), suggesting a mean and significant (*p* < 0.001) value for EE_Intake_ of 3.732 ± 0.558 g/kg BW^0.75^ from 0 to 506 g PKC/kg DM.

The relationship between NDF_Intake_ and PKC_Inclusion_ was influenced by the species (*p* = 0.027). However, the slope (*p* = 0.118) and the intercept (*p* = 0.156) of that relationship did not differ between goats and sheep, resulting in one equation for cattle and another for goats and sheep (Table 2). In cattle, NDF_Intake_ revealed a quadratic increasing association (*p* < 0.001) with PKC_Inclusion_, maximizing the NDF_Intake_ (g/kg BW^0.75^) at 123.5 g PKC/kg DM (Table 2). Goats and sheep exhibited a similar and linear (*p* = 0.033) increasing pattern of NDF_Intake_ as PKC_Inclusion_ increased in the diet (Table 2). Finally, species affected (*p* = 0.024) the relationship between TDN_Intake_ and PKC_Inclusion_, indicating the production of separate regression equations for cattle, goats and sheep (Table 2). Data revealed that TDN_Intake_ (g/kg BW^0.75^) had a linear decreasing pattern (*p* < 0.001) as PKC_Inclusion_ (g/kg DM) increased, irrespective of ruminant species (Table 2).

### 3.2. Dry Matter and Nutrient Digestibility

Data revealed that species affected (*p* < 0.001) the relationship between DM_Dig_ and PKC_Inclusion_ (Figure 3b). Nevertheless, the slope (*p* = 0.125) and the intercept (*p* = 0.382) of that relationship did not differ between cattle and goats. Therefore, one independent equation for cattle and goats and another for sheep were produced. In cattle and goats, DM_Dig_ (g/kg DM) decreased linearly (*p* < 0.001) in response to the increase in PKC_Inclusion_ (g/kg DM; Figure 3b). However, in sheep, DM_Dig_ demonstrated a slight increase (*p* = 0.083) in response to PKC_Inclusion_ (Figure 3b). The species did not influence (*p* = 0.114) the relationship between CP_Dig_ and PKC_Inclusion_, suggesting that one equation is suitable for mathematically describing that relationship. Under that assumption, CP_Dig_ (g/kg DM) showed a linear decrease (*p* = 0.042) as PKC_Inclusion_ (g/kg DM) increased.

The species influenced the slope (*p* = 0.044) and the intercept (*p* = 0.002) of the relationship between EE_Dig_ and PKC_Inclusion_, suggesting independent equations for cattle, goats and sheep (Table 3). The EE_Dig_ (g/kg DM) showed a linear increasing pattern (*p* < 0.056) as PKC_Inclusion_ (g/kg DM) increased, irrespective of species (Table 3). However, the rates of increasing differed (*p* = 0.044) between cattle, goats and sheep. Data revealed that there was an effect of species on the intercept (*p* = 0.062) and slope (*p* = 0.037) of the relationship between NDF_Dig_ and PKC_Inclusion_ (Table 3). However, the exploration of the pairwise statistical comparison tests between species (the *p* values for the slope and intercept pairwise comparison between goats and sheep were 0.583 and 0.224, respectively) revealed that one equation for cattle and another for goats and sheep could be suitable for that relationship (Table 3). In cattle, NDF_Dig_ (g/kg DM) decreased linearly (*p* = 0.083) as PKC_Inclusion_ (g/kg DM) increased, while in goats and sheep, NDF_Dig_ (g/kg DM) showed a linear (*p* = 0.032) increasing pattern.

The exploration of the relationship between TDN_Conc_ and PKC_Inclusion_ revealed an effect (*p* < 0.001) of species on that relationship, resulting in independent equations for cattle, goats and sheep (Table 3). In cattle and goats, TDN_Conc_ (g/kg DM) decreased linearly (*p* < 0.021) as PKC_Inclusion_ (g/kg DM) increased, with a slower rate in cattle than that in goats (Table 3). In contrast, in sheep, TDN_Conc_ (g/kg DM) showed a linear increasing pattern (*p* < 0.001) with the increase in PKC_Inclusion_ (g/kg DM; Table 3).

### 3.3. Performance

The species did not influence (*p* = 0.243) the slope of the relationship between ADG and PKC_Inclusion_ (Table 3). However, the intercept of that relationship was affected (*p* < 0.001). Hence, in this particular case, independent equations for cattle, goats and sheep with a different intercept but a similar slope were produced (Table 3). The equations suggested that the ADG (g/day) at 0 g PKC/kg DM was significantly different (*p* < 0.001) between species. However, ADG (g/day) demonstrated a similar and linear decreasing (*p* = 0.048) pattern as PKC_Inclusion_ (g/kg DM) increased in all the ruminant species explored (Table 3). In contrast, the species affected (*p* < 0.001) the relationship between FE and PKC_Inclusion_. Nevertheless, the slope (*p* = 0.117) and the intercept (*p* = 0.501) of that relationship did not differ between cattle and sheep. Therefore, independent equations, one for goats and another for cattle and sheep, were built. Data revealed a linear increasing effect (*p* = 0.023) of PKC_Inclusion_ on FE (g gain/kg feed) in cattle and sheep (Table 3). However, in goats, FE and PKC_Inclusion_ were related in a linear decreasing manner (*p* = 0.002; Table 3).

## 4. Discussion

Considering the higher price of conventional feedstuffs compared to alternative ones, they can be used in a suitable strategy for optimizing the profitability of ruminant production [4]. Thus, the exploration of the effects of by-products from industrial processes as feedstuffs on nutrient utilization and performance in different ruminants’ species is ever welcome, as their understanding can suggest strategies to reduce costs and optimize animal production in confined systems [27]. In this regard, PKC has been widely explored as a feedstuff in ruminant nutrition, considering the expansion of the clean bio-fuel industry around the world [36], as well as because PKC is compatible with the digestive physiology of the ruminant [37]. As a result, different studies have been conducted, exploring the effects of the dietary inclusion of PKC on nutrient utilization and performance in ruminants, suggesting that these can vary across different species (e.g., cattle, goats and sheep). Nevertheless, this assumption has not been not conclusive until now based on data available in the literature, as published studies were conducted under different production scenarios, whose results in most cases are divergent and contradictory. This limitation can be overcome by gathering data of different studies and analyzing them under a meta-analytic approach, in which inter- and intra-study effects are accounted for in a statistical model [38]. Hence, the major contribution of this study is the use of a meta-analytic approach for testing if dietary PKC_Inclusion_ can produce differential nutritional and performance responses in confined cattle, goats and sheep.

The already mentioned divergent responses in nutrient utilization and performance across individual studies when PKC is explored as a dietary component in cattle, goats and sheep could be defined from a statistical standpoint as heterogeneity across studies. Particularly, this can be attributed to different reasons, including intrinsic divergences in characteristics across studies [22]. This assumption agrees with the significant and high heterogeneity detected across the studies included in the dataset constructed herein. Hence, this condition highlights the importance of considering the study as a random effect, as well as the necessity of conducting a meta-analysis to obtain conclusive results regarding the objective stated.

Publication bias is another important condition to be checked when conducting a meta-analysis to avoid misleading conclusions, ensure the validity and credibility of the results and improve the generalizability of the conclusions [39]. Particularly, an evaluation of publication bias becomes important in meta-analyses, because in some cases, studies with “significant” results have greater probabilities to be published, resulting in a biased estimation of the effects [40,41]. The Egger’s statistical test for asymmetry revealed the absence of publication bias in the current study, which allows for concluding that the dataset constructed could produce reliable results from a meta-analytic approach. This makes sense considering that this meta-analysis explored different publication sources, which significantly decreases over-represented studies with statistically significant findings, optimizes precision in the estimation of size estimates and reduces the inflation of effect sizes estimates [24,42]. Hence, the current models may contribute, from a quantitative standpoint, to elucidating if dietary PKC_Inclusion_ has differential impacts in ruminant species, which is not conclusive from a simple comparison of data derived from different experiments.

Feed intake in ruminants is a complex process to be modeled due to hunger and satiety being affected by diverse factors [43]. Particularly, one could expect an effect of dietary PKC_Inclusion_ on the DM and nutrient intake in cattle, goats and sheep considering the high lipid and fiber contents of PKC across all dietary conditions explored in this study (Table S3). Thus, in light of concomitant differences in the ingestive behavior and the digestive physiology in ruminants, the exploration of the effects of species on the relationship between dietary PKC_Inclusion_ and DM_Intake_ may help to optimize management decision in production systems. This becomes particularly important under confinement scenarios in which PKC is used as a main source, as DM_Intake_ is crucial for defining the net nutrient inputs in ruminants [44].

The data of the current study revealed a negative effect of PKC_Inclusion_ on DM_Intake_, regardless of the ruminant species (e.g., cattle, goat or sheep) in confined systems. This agrees with the classical approach that suggests that a decrease in DM_Intake_ is observed when feedstuff high in fat and low-quality fiber is included in ruminant diets [45]. Additionally, the deleterious effects of increasing levels of other important components in PKC, such as acid detergent lignin (ADL) and ash, along with the concomitant low levels of non-fiber carbohydrates (NFC) (Table S3), may result in reduced ruminal DM fermentation, thereby impairing DM utilization [19,31]. Hence, future studies that explore the effects of all dietary components of PKC on DM utilization from an integrative standpoint could provide valuable insights regarding the better use of this feedstuff in ruminant diets.

Data revealed that the decreasing rate of DM_Intake_ (i.e., slope of the equations) was different between species, being more pronounced in cattle and goats than in sheep. This suggests that increasing levels of PKC can result in more detrimental effects on DM_Intake_ in cattle and goats than in sheep. This makes sense considering the responses observed for DM_Dig_, as PKC_Inclusion_ increased, in which cattle and goats had the greatest decreasing rate, with a slower impact in sheep.

When the equations generated in the present study are adopted for DM intake estimation, a hypothetical inclusion of 100 g/kg DM of PKC would result in a decrease in DM intake of 3.5% in goats or sheep; on the other hand, the same inclusion would result in a decrease in DM intake of 7.8% in cattle. According to literature findings, small ruminants and cattle have differences in ingestive behavior that may impact the DM intake and digestibility of the diet. Boval and Sauvant [12] demonstrated that small ruminants chew faster than cattle, which may allow them to intake slightly more quickly than cattle, explaining the differences between ruminant species in the DM utilization. Also, that difference conforms with fact that goats and sheep tend to be more selective than cattle [46] and also with the differences between small ruminants and cattle in their preferences for feeds [13]. Thus, the quantitative relationships provided herein may assist in the development of future mechanistic models of DM utilization in ruminants. However, the applicability of these results should be restricted to stall-fed animals due to the type of production system possibly influencing the nutritional response in ruminants [9].

An evaluation of the nutritional strategies for decreasing the N excretion in ruminant production systems is an environmental and economic concern, considering its pollutant effects and the energy expenditure of the animal for N excretion [47,48]. According to Souza et al. [49], N excretion in ruminants is influenced by the type of diet. Also, other studies [50] suggested that data on N utilization from one species cannot be applied to other species. Hence, the results reported may provide valuable insights regarding the effects of PKC_Inclusion_ in the diet on N dynamics across different ruminant species.

From the current results, it was observed that species did not influence the relationship of PKC_Inclusion_ with CP_Intake_ and CP_Dig_. This suggests that PKC_Inclusion_ increasing levels may have similar nutritional impacts on CP utilization in cattle, goats and sheep. This pattern interestingly agrees with data obtained by the meta-analysis conducted by Riaz et al. [26], which revealed that increasing CP dietary levels has similar effects on DM_Intake_ decreasing rates in cattle, sheep and goats, when DM_Intake_ is expressed in g/kg metabolic body size. Also, it conforms with the classical theory that suggests that expressing CP requirements for gain as g/kg BW^0.75^ accounts for differences in the body size and metabolic rate between animals, making it easier to compare protein requirements in heterogeneous populations of large [51] and small [52,53] ruminants.

Regarding CP_Dig_, it could be expected that there are similar patterns between ruminant species, as studies gathered in the current dataset involved increasing levels of the same feedstuff (i.e., PKC) with a low CP variation (Table S3), and the type and amount of protein source can significantly influence how efficiently different ruminant species utilize protein [54]. Indeed, this conforms with data reported by Woods et al. [55], who demonstrated that an increase in the feeding levels of diverse concentrate ingredients resulted in a decrease in CP_Dig_, regardless of the ruminant species. This can be stated as it is already known that PKC provides a significant amount of CP in ruminant diets, 106–187 g/kg DM (Table S3) being used in different nutritional scenarios as a protein source [56].

It is widely recognized that increasing levels of fat in the diet of ruminants may have detrimental effects on the digestibility of diverse nutritional components, particularly fiber [57,58]. Hence, the exploration of the impacts of PKC on fat utilization may provide valuable information on the use of this feedstuff in ruminant diets, as PKC is high in lipids [19]. The current study revealed that both EE_Intake_ and EE_Dig_ differed between ruminant species as PKC_Inclusion_ increased. Cattle have the greatest increasing rates for EE_Intake_ in comparison to sheep, but EE_Intake_ was not affected by the PKC_Inclusion_ in goats. Indeed, the patterns for EE_Intake_ conform with the changing rates for EE_Dig_, whose magnitude in cattle was the greatest and not close to that in sheep and goats. This suggests that fat utilization when PKC is included in the diet may differ between ruminant species, in which cattle could tolerate greater fat levels from PKC than goats and sheep. This assumption agrees with Toral et al. [59] findings, who demonstrated that ruminal fatty acid biohydrogenation differs between cows and goats and with the fact that there is a direct relationship between that metabolic process and types and amounts of fatty acids that reach the small intestine [60]. Hence, the ruminal fatty acid biohydrogenation may affect fat digestibility. The aforementioned assumptions can also be linked to the fact that PKC is a high source of lauric and myristic acids [61], and both fatty acids may affect ruminal fermentation, milk production and composition, altering microbial populations in the rumen [62,63]. Thus, the equations produced herein for fat utilization could be potentially used in future studies for exploring the effects of species on the relationship between the dietary inclusion of PKC and the fatty acid composition of ruminant-derived products. This is a clear demand of the meat and milk industry, even considering that it has been demonstrated from early research that the response to fat increasing levels in the diet may vary across cattle, goats and sheep, resulting in different contents of protein and fatty acid profiles [64].

It has been demonstrated that fat intake and fiber digestibility have a nutritionally opposite relationship in the ruminant [65]. This assumption agrees with the results obtained, in which an increase in EE_Intake_ resulted in a concomitant decrease in the NDF_Dig_ in cattle. Interestingly, this assumption can also be adopted for small ruminants, due to PKC_Inclusion_ increases not affecting EE_Intake_ in goats, while it slightly increased in sheep. As a result, NDF_Dig_ increased in sheep and cattle. This constitutes a novel result, suggesting, for the first time, that the interaction between fiber and fat when PKC is the main dietary constituent may differ between cattle and small ruminants. Hence, and considering that fatty acid biohydrogenation differs between cattle and small ruminants [59], the current data fit with the already known relationship between fibrolytic microorganisms and the fatty acid biohydrogenation in the rumen [66].

The equations obtained in the current study revealed different patterns for NDF and TDN utilization among cattle, goats and sheep when dietary PKC_Inclusion_ was increased. First, NDF_Intake_ increased as PKC_Inclusion_ increased, concomitantly with a TDN_Intake_ decrease, regardless of the ruminant species. These responses are expected as PKC is a significant NDF source in ruminant diets, thereby negatively impacting the intake of TDN in the diet [19]. However, the novelty of the results reported herein suggests that cattle, goats and sheep may utilize NDF and TDN in different ways when PKC is included as the main dietary feedstuff. This was confirmed by the observed significant effects of species on the relationships of NDF and TDN intake and digestibility with PKC_Inclusion_—particularly, the fact that goats and sheep apparently utilize fiber in a similar way compared to cattle. These patterns agree with the already known significant differences that exist in the digestive tract [67] and in the rumen microbiome composition [68] between ruminant species. Indeed, the basic four-chambered stomach structure (rumen, reticulum, omasum and abomasum) can vary in size and proportions across ruminants [69]. Also, goats and sheep have different rumen microbial populations compared to cattle [70]. All these facts may impact the ability of the ruminant to break down and digest fiber. Thus, the data reported revealed novel insights regarding how fiber is used in cattle, goats and sheep when PKC is included as the main feedstuff in confined ruminant diets.

Considering the abundance and lower costs of PKC compared to traditional feedstuffs, different studies have been conducted, exploring its effects on ruminant performance in confined cattle, goats and sheep [4,5,10], but with contradictory responses. These differences could be associated with heterogeneity in conditions across studies, which may affect the intricate and complex relationships among intake, digestibility and performance in ruminants [69]. The disparities among studies could be overcome by using a meta-analytical approach, which quantifies the aforementioned inter- and intra-study variation with a significant enhancement in the inference range and the power of predictions in quantitative models [21].

The data of this study reveal that ADG differed between species at 0 g PKC/kg DM, which is expected, as this variable is affected by feed intake, regardless of diet [71]. However, and interestingly, the three ruminant species have the same decreasing rate for this productive variable, as PKC_Inclusion_ increased in the diet. The decrease in the ADG rate makes sense, as regardless of species and sex, ruminants tend to have a lower muscle: fat ratio as they approach mature weight [72,73]. However, the novelty of this study lies in the fact that data suggested for the first time that PKC may have similar impacts on ADG in cattle, goats and sheep, which may be valuable information considering the economic importance of ADG optimization in ruminant production [74].

Feed efficiency (FE) is considered during the evaluation of the economic suitability of a ruminant production system, as it explores how the animal converts feed into desired products (e.g., milk, meat, wool) from a quantitative standpoint [75]. Hence, FE can be used for optimizing feeding costs, which may be beneficial for animal profitability, since feeding supports approximately 65% of animal production costs [76]. The data of the current study revealed that PKC_Inclusion_ has differential effects on FE in cattle, sheep and goats.

Although the equations had revealed differences in FE between species, at 277.4 g/kg of PKC in the diet, the ruminants present a similar performance (i.e., 121.8 g gain/kg feed). The differences in the patterns of the observed changing PKC_Inclusion_ levels could be due to nutrient utilization dynamics differing between ruminant species [77] and the direct relationship between nutrient metabolism and FE in ruminants [78]. Hence, the data suggest that nutritional strategies considering PKC as the main feedstuff should be discriminated by the ruminant’s species in confined systems in order to enhance animal profitability. However, the potential deleterious impacts of PKC on performance, regardless of ruminant species, suggest that quantitative approaches beyond the inclusion level may be desirable for promoting nutritional strategies to enhance the DM digestibility of PKC, thereby positively affecting ADG and FE. Such strategies may include conducting a meta-analysis that accounts for species effects while discriminating among PKC processing treatments—such as fermentation [79], enzymatic hydrolysis and steam pretreatment [56]—which the literature indicates to be suitable for improving the digestibility of abundant polysaccharides in PKC, such as β-mannan [80]. These research directions could provide valuable insights into the use of this feedstuff in ruminant production.

## 5. Conclusions

The data of the current meta-analysis provide an overview on the relationship of PKC_Inclusion_ with nutrient utilization and performance in different confined ruminant species. The data revealed that PKC_Inclusion_ has differential effects on the DM intake and digestibility of most nutritional components in cattle, goats and sheep. Regarding performance, the data suggest that those ruminant species may have similar average daily gain decreasing rates but differences in feed efficiencies. Additionally, considering that the equations reported herein treat PKC_Inclusion_ as a continuous variable, these can be used in future nutritional models to allow for the better use of alternative feedstuffs. This strategy may result in the designing of more sustainable nutritional approaches including by-products derived from diverse worldwide production systems. Hence, future studies could apply meta-analysis to investigate the dietary effects of PKC across different ruminant species while accounting for production systems (e.g., grazing versus confined), differences between dairy and beef breeds and the impacts on milk yield and composition, final body weight, as well as carcass traits and meat characteristics. This could expand the understanding of the nutritional effects of PKC in ruminants, beyond the feeding context.

## Figures and Tables

**Table 1:** Summary of the dataset developed for exploring the effects of species on the dietary relationship of palm kernel cake (PKC) inclusion with nutrient utilization and performance in confined cattle, goats and sheep.

Parameter	Mean	Standard Deviation	Minimum	Maximum
Cattle				
PKC inclusion in the diet (g/kg DM)	132.9	156.9	0.00	890.0
Mean BW (kg)	365.7	140.3	149.4	566.8
Daily feed intake (g/kg BW^0.75^)				
Dry matter	111.7	29.27	54.64	193.2
Crude protein	12.98	4.47	3.13	24.97
Ether extract	4.77	1.91	2.34	9.22
Neutral detergent fiber	45.89	16.67	20.19	84.83
Total digestible nutrients	75.84	19.39	42.07	120.3
Digestibility (g/kg DM)				
Dry matter	641.8	74.26	440.0	762.0
Crude protein	673.5	89.85	516.1	853.0
Ether extract	761.6	92.33	576.4	953.4
Neutral detergent fiber	582.1	106.5	357.6	746.1
Total digestible concentration	683.9	64.09	571.6	798.6
Performance				
Average daily gain (g/day)	933.2	341.7	141.1	1540
Feed efficiency (g gain/kg feed)	135.5	30.1	67.70	249.4
Goats				
PKC inclusion in the diet (g/kg DM)	137.3	114.4	0.00	506.3
Mean BW (kg)	30.2	15.2	7.48	60.0
Daily feed intake (g/kg BW^0.75^)				
Dry matter	77.48	21.95	45.21	135.7
Crude protein	11.93	4.87	4.94	27.83
Ether extract	3.61	1.22	1.92	6.02
Neutral detergent fiber	33.22	9.79	13.77	48.85
Total digestible nutrients	55.55	16.36	26.79	78.89
Digestibility (g/kg DM)				
Dry matter	674.4	55.50	545.6	756.2
Crude protein	693.7	77.96	521.0	862.0
Ether extract	867.8	81.76	584.0	949.2
Neutral detergent fiber	555.4	106.6	285.0	730.3
Total digestible concentration	675.6	61.87	576.5	771.0
Performance				
Average daily gain (g/day)	77.0	60.3	10.2	219.0
Feed efficiency (g gain/kg feed)	123.5	70.7	20.02	226.0
Sheep				
PKC inclusion in the diet (g/kg DM)	175.8	173.0	0.00	650.0
Mean BW (kg)	26.4	6.97	10.3	36.9
Daily feed intake (g/kg BW^0.75^)				
Dry matter	82.35	19.32	41.00	119.7
Crude protein	12.58	4.30	6.07	21.26
Ether extract	3.26	1.09	1.50	5.66
Neutral detergent fiber	47.82	12.86	22.24	78.64
Total digestible nutrients	66.39	19.29	36.91	106.6
Digestibility (g/kg DM)				
Dry matter	700.1	87.55	478.0	838.0
Crude protein	732.4	87.83	478.9	860.6
Ether extract	872.7	45.92	799.0	945.8
Neutral detergent fiber	686.3	86.20	513.0	813.2
Total digestible concentration	663.9	70.34	577.7	792.0
Performance				
Average daily gain (g/day)	124.1	56.70	16.07	185.0
Feed efficiency (g gain/kg feed)	107.8	44.20	26.34	178.6

**Table 2:** Quantitative effects of species on the relationship between dietary palm kernel cake inclusion (g/kg DM) and nutrient intake (g/kg BW^0.75^) in confined ruminants.

Species	Equations ^a,b^	N ^c^	Statistics ^d^			*p* Value (Species) ^e^		
			CCC	R^2^	RMSE	Intercept	Linear Slope	Quadratic Slope
Cattle, goats and sheep	CP_Intake_ = 14.26 (±0.894 **) − 0.0124 (±0.00181 **) × PKC	104	0.942	0.899	1.49	0.765	0.807	-
Cattle	EE_Intake_ = 3.705 (±0.547 **) + 0.0120 (±0.00264 **) × PKC − 0.00003 (±9.578 × 10^−6^ **) × PKC^2^	42	0.958	0.922	0.54	0.462	0.013	0.075
Goats	EE_Intake_^f^ = 3.732 (±0.558 **)	19	-	-	-			
Sheep	EE_Intake_ = 2.846 (±0.452 **) + 0.00345 (±0.00069 **) × PKC	26	0.951	0.907	0.33			
Cattle	NDF_Intake_ = 43.82 (±4.74 **) + 0.0642 (±0.0199 **) × PKC − 0.00026 (±0.00007 **) × PKC^2^	43	0.968	0.939	4.03	0.143	0.578	0.027
Goats and sheep	NDF_Intake_ = 37.76 (±3.079 **) + 0.0155 (±0.00606 **) × PKC	74	0.930	0.879	4.98			
Cattle	TDN_Intake_ = 86.15 (±5.503 **) − 0.0841 (±0.0119 **) × PKC	39	0.946	0.904	6.15	0.106	0.024	-
Goats	TDN_Intake_ = 69.72 (±8.841 **) − 0.102 (±0.0202 **) × PKC	14	0.903	0.821	7.20			
Sheep	TDN_Intake_ = 68.79 (±7.266 **) − 0.0417 (±0.0139 **) × PKC	25	0.966	0.940	5.47			

^a^ General form of mathematical equations: Linear relationship (L): Y = B_0_ + B_1_ × PKC, quadratic relationship (Q): Y = B_0_ + B_1_ × PKC + B_2_ × PKC^2^, in which PKC = palm kernel cake inclusion level. CP_Intake_ = crude protein intake, EE_Intake_ = ether extract intake, NDF_Intake_ = neutral detergent fiber intake and TDNIntake = total digestible nutrients intake. Values within parentheses correspond to the standard error of the parameters in the equation, with ** *p* < 0.05. ^b^ When the quadratic component was not significant (*p* > 0.10), it was removed from the model, and species effects were not tested for that component (-). ^c^ N = total number of treatment means used for fitting the equation after data depuration (remotion of outliers and influence values). ^d^ CCC = concordance correlation coefficient; R^2^ = coefficient of determination; RMSE = root mean square error. ^e^ Test for species’ effect assuming a significant level equal to 0.10. ^f^ There was no effect of dietary PKC inclusion on EE_Intake_ in goats. Hence, a mean value for EE_Intake_ was reported for that species.

**Table 3:** Quantitative effects of species on the relationship of dietary palm kernel cake inclusion (g/kg DM) with nutrient digestibility (g/kg DM) and performance in confined ruminants.

Species	Equations ^a,b^	N ^c^	Statistics ^d^			*p* Value (Species) ^e^	
			CCC	R^2^	RMSE	Intercept	Linear Slope
	Digestibility (g/kg DM)						
Cattle, goats and sheep	CP_Dig_ = 701.3 (±15.45 **) − 0.0560 (±0.0272 **) × PKC	132	0.934	0.882	30.22	0.460	0.114
Cattle	EE_Dig_ = 717.6 (±23.09 **) + 0.313 (±0.0580 **) × PKC	48	0.919	0.857	35.99	0.002	0.044
Goats	EE_Dig_ = 829.9 (±31.48 **) + 0.116 (±0.0593 *) × PKC	27	0.920	0.881	28.96		
Sheep	EE_Dig_ = 852.1 (±38.16 **) + 0.142 (±0.0688 *) × PKC	19	0.922	0.859	19.37		
Cattle	NDF_Dig_ = 581.0 (±31.08 **) − 0.126 (±0.0796 *) × PKC	48	0.885	0.804	50.94	0.062	0.037
Goats and sheep	NDF_Dig_ = 586.5 (±25.63 **) + 0.125 (±0.0574 **) × PKC	75	0.937	0.888	42.03		
Cattle	TDN_Conc_ = 693.5 (±23.50 **) − 0.0930 (±0.03882 **) × PKC	28	0.957	0.920	16.55	0.191	<0.001
Goats	TDN_Conc_ = 694.2 (±28.48 **) − 0.161 (±0.0496 **) × PKC	17	0.966	0.935	16.28		
Sheep	TDN_Conc_ = 632.1 (±27.93 **) + 0.122 (±0.0335 **) × PKC	22	0.967	0.939	17.41		
	Performance						
Cattle	ADG (g/day) = 961.7 (±79.10 **) − 0.157 (±0.0783 *) × PKC	46	0.949	0.908	104.5	<0.001	0.243
Goats	ADG (g/day) = 99.26 (±104.8 **) − 0.157 (±0.0783 *) × PKC	24	0.953	0.914	18.57		
Sheep	ADG (g/day) = 129.1 (±112.6 **) − 0.157 (±0.0783 *) × PKC	24	0.982	0.967	10.58		
Cattle and sheep	FE (g gain/kg feed) = 113.4 (±12.03 **) + 0.0302 (±0.0174 **) × PKC	66	0.938	0.886	13.07	0.431	0.001
Goats	FE (g gain/kg feed) = 139.2 (±17.80 **) − 0.0628 (±0.0192 **) × PKC	24	0.968	0.940	17.33		

^a^ General form of mathematical equations: Linear relationship (L): Y = B_0_ + B_1_× PKC, in which PKC = palm kernel cake inclusion level, CP_Dig_ = crude protein digestibility, EE_Dig_ = ether extract digestibility, NDF_Dig_ = neutral detergent fiber digestibility, TDN_Dig_ = total digestible nutrients digestibility, ADG = average daily gain (g/day), and FE = feed efficiency (g gain/kg feed). Values within parentheses correspond to the standard error of the parameters in the equation; ** *p* < 0.05 and * 0.05 ≤ *p* < 0.10. ^b^ The quadratic effect of PKC inclusion on digestibility and performance variables was tested, but it was not significant (*p* > 0.10) in the relationships explored. ^c^ N = total number of treatment means used for fitting the equation after data depuration (remotion of outliers and influence values). ^d^ CCC = concordance correlation coefficient; R^2^ = coefficient of determination; RMSE = root mean square error. ^e^ Test for species’ effect assuming a significant level equal to 0.10.

## Data Availability

The data presented in this study are available upon reasonable request from the corresponding author.

## Supplementary Materials

The following supporting information can be downloaded at: https://www.mdpi.com/article/10.3390/ani15182764/s1, Table S1: Description of the variables extracted from the studies for conducting the meta-analysis; Table S2: Mean values of nutritional components of experimental diets used in the studies included in the meta-analysis; Table S3: Descriptive statistics of the chemical composition of palm kernel cake used in the studies with cattle, goats and sheep. Refs. [81,82,83,84,85,86,87,88,89,90,91,92,93,94,95,96,97,98,99,100,101,102,103,104,105,106,107,108,109,110,111,112,113,114,115,116,117,118,119,120,121,122,123,124,125,126] are cited in Table S1.
