# Supplementary material for: Effects of Palm Kernel Cake on Nutrient Utilization and Performance in Confined Cattle, Sheep and Goats: A Comparative Meta-Analytical Approach
Source: Animals (Basel). 2025 Sep 22;15(18):2764. doi: 10.3390/ani15182764 (PMC12466351; doi:10.3390/ani15182764)
Supplement: Supplementary file 1 [file animals-15-02764-s001.zip › Table S1.pdf]

Table S1. Description of the variables extracted from the studies for conducting the meta-analysis

No	Study	Palm kernel cake inclusion levels (g/kg DM the diet)	Specie	Intake (g/kg $BW^{0.75}$)					Digestibility (g/kg DM)					Performance	
				DM	CP	EE	NDF	TDN	DM	CP	EE	NDF	TDN	ADG (g/day)	FE (g gain/kg feed)
1	Abdullah and Hutagalung, 1988	890	Cattle	+										+	+
2	Correia et al., 2011	0 and 160	Cattle	+	+	+	+	+	+	+	+	+			
3	Fereira et al., 2012	0, 70, 141, 211, and 281	Cattle	+	+	+	+	+	+	+	+	+			
4	Maciel et al., 2012	0, 119, 229, and 342	Cattle	+	+	+	+	+	+	+	+	+	+	+	+
5	Cruz, 2013	0, 70, 140, and 210	Cattle	+	+	+	+	+	+	+	+	+	+		
6	Cunha et al., 2013	0, 113, 228, and 342	Cattle	+	+	+	+	+	+	+	+	+			
7	Santana Filho, 2013	0, 70, 140, and 210	Cattle											+	+
8	Pimentel et al., 2015	0, 43, 92, and 138	Cattle	+			+	+							
9	Martins, 2016	0, 61, 127, and 187	Cattle	+	+	+	+	+	+	+	+	+	+		
10	Sani et al., 2017	0, 50, 100, 150, and 200	Cattle											+	+
11	Pimentel et al., 2018	0, 43, 92, and 138	Cattle		+	+			+	+	+	+	+	+	
12	Sani, 2018	0, 50, 100, 150, and 200	Cattle						+	+	+	+			
13	Cruz et al., 2018	0, 70, 140, and 210	Cattle	+	+	+	+		+	+	+	+		+	+
14	Hussein et al., 2018	0, 80, and 159	Cattle	+						+	+			+	+
15	Iqbal et al., 2019	0, 100, and 200	Cattle											+	
16	Santos et al., 2019	0, 80, 160, and 240	Cattle	+	+	+	+	+	+	+	+	+	+	+	+
17	Lisboa et al., 2021	0, 80, 160, and 240	Cattle	+	+	+	+	+	+	+	+	+	+	+	+
18	Sani et al., 2021	0, 50, 100, 150, and 200	Cattle	+										+	+
19	Abreu et al., 2024	0, 100, 200, and 300	Cattle	+	+	+	+	+	+	+	+	+	+	+	+
20	Gunawan et al., 2023	0, 76, and 126	Cattle	+										+	+
21	Carvalho et al., 2004	0, 94, and 188	Goat	+			+								
22	Silva et al., 2005a	0, 94, and 188	Goat						+	+	+	+			
23	Silva et al., 2005b	0, 94, and 188	Goat	+	+	+	+	+					+		
24	Chanjula et al., 2010a	100, 160, 223, 303, and 378	Goat	+					+	+		+			
25	Chanjula et al., 2010b	128, 195, 113, 172, 115, and 151	Goat	+					+	+	+	+			
26	Chanjula and Pengnoo, 2012	0, 32, 67, 97, and 128	Goat	+					+	+	+	+			
27	Abubakr et al., 2013a	0 and 506	Goat	+					+	+	+	+			
28	Abubakr et al., 2013b	0 and 784	Goat	+										+	+

29	Rahman et al., 2013b	0, 40, 95, and 141	Goat	+	+		+		+	+		+		+	+
30	Rahman et al., 2013a	0 and 249	Goat	+	+		+		+	+		+		+	+
31	Oliveira et al., 2017	0, 70, 140, and 210	Goat	+			+								
32	Ribeiro et al., 2018	0, 70, 140, and 210	Goat	+	+	+	+	+	+	+	+	+	+	+	+
33	Olawoye et al., 2020	0, 89, 166, 239, and 267	Goat	+										+	+
34	Silva et al., 2020	0, 120, 240, and 360	Goat	+	+	+	+	+	+	+	+	+	+	+	+
35	Rodrigues et al., 2021	0, 120, 240, and 360	Goat	+	+	+	+		+	+	+	+	+	+	+
36	Ferreira et al., 2022	0, 80, 160, and 240	Goat	+	+	+	+	+	+	+	+	+	+		
37	Arief et al., 2023	0, 100, 200, and 300	Goat	+	+				+	+					
38	Arief et al., 2024	0, 150, 250, and 350	Goat	+	+				+	+					
39	Lakshmi and Krishna, 1995	0, 50, 100, and 150	Sheep	+	+					+		+			
40	Umunna et al., 1994	0, 200, 350, 500, and 650	Sheep	+					+	+				+	+
41	Carvalho et al., 2006	0 and 160	Sheep	+			+								
42	Costa et al., 2010	100, 200, 300, and 400	Sheep	+			+		+			+			
43	Bringel et al., 2011	0, 200, 400, 600, and 800	Sheep	+	+	+	+	+	+	+	+	+	+		
44	Macome et al., 2011	0, 65, and 130	Sheep	+	+	+	+	+					+	+	+
45	Nunes et al., 2011	0, 135, and 195	Sheep	+	+	+	+	+	+	+	+	+			
46	Macome et al., 2012	0, 65, 130, and 195	Sheep	+			+	+					+	+	+
47	Visoná-Oliveira et al., 2015	0, 75, 150, and 225	Sheep	+	+	+	+	+	+	+		+	+		
48	Pinho et al., 2016	0, 150, 300, 450, and 600	Sheep	+			+								
49	Santos et al., 2016	0, 75, 150, 225, and 300	Sheep	+	+	+	+	+	+	+	+	+	+	+	+
50	Omotoso et al., 2021	0, 50, 100, and 250	Sheep	+	+	+	+		+	+	+	+		+	+
51	Castro et al., 2023	0 and 176	Sheep	+	+	+	+		+	+	+	+		+	+

+ = variables included in the study

References used for meta-analysis

1. Abdullah, N.; Hutagalung, R.I. Rumen Fermentation, Urease Activity and Performance of Cattle given Palm Kernel Cake-Based Diet. *Anim Feed Sci Technol* **1988**, *20*, 79–86, doi:10.1016/0377-8401(88)90129-0.
2. Correia, B.R.; Oliveira, R.L.; Jaeger, S.M.P.L.; Bagaldo, A.R.; Carvalho, G.G.P.; Oliveira, G.J.C.; Lima, F.H.S.; Oliveira, P.A. Consumo, Digestibilidade e pH Ruminal de Novilhos Submetidos a Dietas com Tortas Oriundas da Produção do Biodiesel em Substituição ao Farelo de Soja. *Arq Bras Med Vet Zootec* **2011**, *63*, 356–363, doi:10.1590/S0102-09352011000200013.
3. Fereira, A.C.; Lopes, R.O.; Regina, A.B.; Giordano-Pinto, G.C.; Nunes-Vaz, R.S.; Andrade, P.O. Intake, Digestibility and Intake Behaviour in Cattle Fed Different Levels of Palm Kernel Cake. *Rev MVZ Córdoba* **2012**, *17,* 3105–3112.
4. Maciel, R.P.; Neuman, J.; Neiva, M.; Araujo, V.L.; Fagner, O.; Cunha, R.; Paiva, J.; Restle, J.; Mendes, C.Q.; Lôbo, N.B. Intake, Nutrient Digestibility and Performance of Dairy Heifers Fed Diets Containing Palm Kernel Cake. *Rev Bras Zootec* **2012**, *41*, 698–706, doi: https://doi.org/10.1590/S1516-35982012000300033.
5. Cruz, C.H. Desempenho Bioeconômico de Novilhos Submetidos a Dietas com Níveis de Torta de Dendê, Oriunda da Produção do Biodiesel. MSc Thesis, Federal University of Bahia: Salvador - BA, 2013.
6. Cunha, O.F.R.; Neiva, J.N.M.; Maciel, R.P.; Restle, J.; Araújo, V.L.; Paiva, J.; Miotto, F.R.C. Palm (*Elaeis Guineensis* L.) Kernel Cake in Diets for Dairy Cows. *Semin Cienc Agrar* **2013**, *34*, 445–454, doi:10.5433/1679-0359.2013v34n1p445.
7. Santana Filho, N.B. Características de Carcaça e Qualidade da Carne de Tourinhos Nelore Submetidos a Dietas com Níveis de Torta de Dendê, Oriunda da Produção do Biodiesel. MSc Thesis, Federal University of Recôncavo da Bahia: Cruz das Almas - BA, 2013.
8. Pimentel, L.R.; Silva, F.F.; Silva, R.R.; Schio, A.R.; Oliveira Rodrigues, E.S.; Oliveira, P.A. Comportamento Ingestivo de Vacas Lactantes Alimentadas com Níveis de Torta de Dendê na Dieta. *Acta Sci Anim Sci* **2015**, *37*, 83–89, doi:10.4025/actascianimsci.v37i1.23391.
9. Martins, L.F.D. Torta de Dendê em Dietas para Vacas Lactantes Confinadas, PhD Dissertation, Southwest Bahia State University: Itapetinga - BA, 2016.
10. Sani, R.T.; Lamidi, O.S.; Dung, D.D.; Hassan, M.R. Performance of Yearling Bunaji Bulls Fed Diets Containing Graded Level of Palm Kernel Cake. *Nigerian J Anim Sci* **2017**, *2017*, 235–246.
11. Pimentel, L.R.; Da Silva, F.F.; Silva, R.R.; Porto, A.F.; Costa, E.G.L.; Schio, A.R.; De Souza, D.D.; Rodrigues, E.S.D.O.; Da Silva, G.M.; Menezes, M.D.A. Production Performance of Crossbred Dairy Cows Fed Palm Kernel Cake in Feedlots. *Semin Cienc Agrar* **2018**, *39*, 2103–2112, doi:10.5433/1679-0359.2018v39n5p2103.
12. Sani, R.T.; Lamidi, O.S.; Dung, D.D.; Hassan, M.R. Nutrient Digestibility and Nitrogen Balance in Yearling Bunaji Bulls Fed Diets Containing Graded Levels of Palm Kernel Cake. *Niger J Anim Prod* **2018**, *12,* 720–723.
13. Cruz, C.H.; Silva, T.M.; Santana Filho, N.B.; Leão, A.G.; Ribeiro, O.L.; Carvalho, G.G.P.; Bezerra, L.R.; Oliveira, R.L. Effects of Palm Kernel Cake (*Elaeis Guineensis*) on Intake, Digestibility, Performance, Ingestive Behaviour and Carcass Traits in Nellore Bulls. *J Agric Sci* **2018**, *156*, 1145–1152, doi:10.1017/S0021859618001168.
14. Hussein, A.M.; Mousa, S.A.; Fahmy, K.N.; Ismail, E.Y. Influence of Dietary Inclusion of Palm Kernel Meal (PKM) and Live Yeast on Growth Performance, Rumen Fermentation Parameter, Nutrient Digestibility and Blood Biochemical Indices in Beef Calves. *J Egypt Vet Med Assoc* **2018**, *78*, 609–621.
15. Iqbal, Z.; Rashid, M.A.; Pasha, T.N.; Bhatti, J.A. Effect of Feeding Varying Levels of Palm Kernel Cake on Production Performance and Blood Metabolites of Lactating Crossbred Dairy Cattle. *J Anim Plant Sci* **2019**, *29*, 419–424.

16. Santos, L. V.; Silva, R.R.; Silva, F.F.; Silva, J.W.D.; Barroso, D.S.; Silva, A.P.G.; Souza, S.O.; Santos, M.C. Increasing Levels of Palm Kernel Cake (*Elaeis Guineensis* Jacq.) in Diets for Feedlot Cull Cows. *Chil J Agric Res* **2019**, *79*, 628–635, doi:10.4067/S0718-58392019000400628.
17. Lisboa, M.; Silva, R.R.; Silva, F.F.; Carvalho, G.G.P.; Silva, J.W.D.; Paixão, T.R.; Silva, A.P.G.; Carvalho, V.M.; Santos, L.V.; Conceição Santos, M.; et al. Replacing Sorghum with Palm Kernel Cake in the Diet Decreased Intake without Altering Crossbred Cattle Performance. *Trop Anim Health Prod* **2021**, *53*, doi:10.1007/s11250-020-02460-x.
18. Sani, R.T.; Okin-Aminu, H.O.; Idowu, W.; Achi, N.P.; Ahmed, S.A.; Bello, S.S. Feed Intake, Rumen Metabolite and Some Blood Parameters of Yearling Bunaji Bulls Fed Graded Levels of Palm Kernel Cake. *Niger J Anim Prod* **2021**, *48*, 311–327, doi:10.51791/njap.v48i5.3218.
19. Abreu, G.; Silva, F.F.; Azevêdo, J.A.G.; Silva, J.W.D.; Paixão, T.R.; Costa, G.D.; Santos, L.V.; Silva, A.P.G.; Carvalho, G.G.P.; Lima, D.M.; et al. Effect of Palm Kernel Cake Inclusion on Intake, Digestibility, Nitrogen Balance, Feeding Behavior, and Weight Gain of Feedlot Heifers. *Rev Bras Zootec* **2024**, *53*, doi:10.37496/rbz5320230132.
20. Gunawan; Winarti, E.; Sofyan, A.; Putridinanti, A.D.; Andarwati, S.; Noviandi, C.T.; Agus, A.; Harper, K.J.; Poppi, D.P. Improving Growth Rates of Ongole Crossbred Bulls by Formulation and Level of Supplement of By-Products. *Anim Prod Sci* **2023**, *64*, doi:10.1071/AN23229.
21. Carvalho, G.G.P.; Pires, A.J.V.; Silva, F.F.; Veloso, C.M.; Silva, R.R.; Silva, H.G.O.; Bonomo, P.; Mendonça, S.S. Comportamento Ingestivo de Cabras Leiteiras Alimentadas com Farelo de Cacau ou Torta de Dendê. *Pesqui Agropecu Bras* **2004**, *39*, 919–925, doi:10.1590/S0100-204X2004000900012.
22. Silva, H.G.O.; Pires, A.J.V.; Silva, F.F.; Veloso, C.M.; Carvalho, G.G.P.; Cezário, A.S.; Santos, C.C. Digestibilidade Aparente de Dietas Contendo Farelo de Cacau ou Torta de Dendê em Cabras Lactantes. *Pesqui Agropecu Bras* **2005**, *40*, 405–411, doi:10.1590/S0100-204X2005000400013.
23. Silva, H.G.O.; Pires, A.J.V.; Silva, F.F.; Veloso, C.M.; Carvalho, G.G.P.; Cezário, A.S.; Santos, C.C. Farelo de Cacau (*Theobroma Cacao* L.) e Torta de Dendê (*Elaeis Guineensis*, Jacq) na Alimentação de Cabras em Lactação: Consumo e Produção de Leite. *Rev Bras Zootec* **2005**, *34*, 1786–1794, doi:10.1590/S1516-35982005000500040.
24. Chanjula, P.; Mesang, A.; Pongprayoon, S. Effects of Dietary Inclusion of Palm Kernel Cake on Nutrient Utilization, Rumen Fermentation Characteristics and Microbial Populations of Goats Fed Paspalum Plicatulum Hay-Based Diet. *Songklanakarin J Sci Technol* **2010**, *32*, 527–536.
25. Chanjula, P.; Siriwathananukul, Y.; Lawpetchara, A. Effect of Feeding Rubber Seed Kernel and Palm Kernel Cake in Combination on Nutrient Utilization, Rumen Fermentation Characteristics, and Microbial Populations in Goats Fed on Briachiaria Humidicola Hay-Based Diets. *Asian-Australas J Anim Sci* **2010**, *24*, 73–81, doi:10.5713/ajas.2011.10171.
26. Chanjula, P.; Pengnoo, A. Influence of Replacing Soybean Meal with Yeast Fermented Palm Kernel Cake in Concentrate on Nutrient Utilization and Rumen Fermentation Characteristics in Goats. In Proceedings of the *The 1st International Conference on Animal Nutrition and Environment*; Khon Kaen, Thailand, 2012; pp. 487–490.
27. Abubakr, A.R.; Alimon, A.R.; Yaakub, H.; Abdullah, N.; Ivan, M. Digestibility, Rumen Protozoa, and Ruminal Fermentation in Goats Receiving Dietary Palm Oil by-Products. *J Saudi Soc Agric Sci* **2013**, *12*, 147–154, doi:10.1016/j.jssas.2012.11.002.
28. Abubakr, A.R.; Alimon, A.R.; Yaakub, H.; Abdullah, N.; Ivan, M. Growth, Nitrogen Metabolism and Carcass Composition of Goats Fed Palm Oil by-Products. *Small Rumin Res* **2013**, *112*, 91–96, doi:10.1016/j.smallrumres.2012.11.003.
29. Rahman, M.M.; Abdullah, R.B.; Wan Embong, W.K.; Nakagawa, T.; Akashi, R. Effect of Palm Kernel Cake as Protein Source in a Concentrate Diet on Intake, Digestibility and Live Weight Gain of Goats Fed Napier Grass. *Trop Anim Health Prod* **2013**, *45*, 873–878, doi:10.1007/s11250-012-0300-4.
30. Rahman, M.M.; Abdullah, R.B.; Wan Khadij, W.E.; Nakagawa, T.; Akashi, R. Feed Intake, Digestibility and Growth Performance of Goats Offered Napier Grass Supplemented with

Molasses Protected Palm Kernel Cake and Soya Waste. *Asian J Anim Vet Adv* **2013**, *8*, 527–534, doi:10.3923/ajava.2013.527.534.
31. Oliveira, R.L.; Carvalho, G.G.P.; Oliveira, R.L.; Tosto, M.S.L.; Santos, E.M.; Ribeiro, R.D.X.; Silva, T.M.; Correia, B.R.; Rufino, L.M.A. Palm Kernel Cake Obtained from Biodiesel Production in Diets for Goats: Feeding Behavior and Physiological Parameters. *Trop Anim Health Prod* **2017**, *49*, 1401–1407, doi:10.1007/s11250-017-1340-6.
32. Ribeiro, R.D.X.; Oliveira, R.L.; Oliveira, R.L.; Carvalho, G.G.P.; Medeiros, A.N.; Correia, B.R.; Silva, T.M.; Bezerra, L.R. Palm Kernel Cake from the Biodiesel Industry in Diets for Goat Kids. Part 1: Nutrient Intake and Utilization, Growth Performance and Carcass Traits. *Small Rumin Res* **2018**, *165*, 17–23, doi:10.1016/j.smallrumres.2018.05.013.
33. Olawoye, S.O.; Okeniyi, F.A.; Adeloye A.A.; Alabi, O.O.; Shoyombo, A.J.; Animashahun, R.A.; Yousuf, M.B. Effects of Formulated Concentrate and Palm Kernel Cake Supplementation on Performance Characteristics of Growing West African Dwarf (WAD) Goat Kids. *Niger J Anim Sci* **2020**, *22*, 287–295.
34. Silva, L.O.; Carvalho, G.G.P.; Tosto, M.S.L.; Lima, V.G.O.; Cirne, L.G.A.; Pina, D.S.; Santos, S.A.; Rodrigues, C.S.; Ayres, M.C.C.; Azevedo, J.A.G. Digestibility, Nitrogen Metabolism, Ingestive Behavior and Performance of Feedlot Goats Fed High-Concentrate Diets with Palm Kernel Cake. *Livest Sci* **2020**, *241*, 104226, doi:10.1016/j.livsci.2020.104226.
35. Rodrigues, T.C.G.C.; Santos, S.A.; Cirne, L.G.A.; Santos-Pina, D.; Alba, H.D.R.; Araújo, M.L.G.M.L.; Silva, W.P.; Oliveira Nascimento, C.; Rodrigues, C.S.; Tosto, M.S.L.; et al. Palm Kernel Cake in High-Concentrate Diets for Feedlot Goat Kids: Nutrient Intake, Digestibility, Feeding Behavior, Nitrogen Balance, Blood Metabolites, and Performance. *Trop Anim Health Prod* **2021**, *53*, 454, doi:10.1007/s11250-021-02893-y.
36. Ferreira, F.G.; Leite, L.C.; Alba, H.D.R.; Pina, D.S.; Santos, S.A.; Tosto, M.S.L.; Rodrigues, C.S.; Lima-Júnior, D.M.; Oliveira, J.S.; Freitas Júnior, J.E.; et al. Palm Kernel Cake in Diets for Lactating Goats: Intake, Digestibility, Feeding Behavior, Milk Production, and Nitrogen Metabolism. *Animals* **2022**, *12*, 2323, doi:10.3390/ani12182323.
37. Arief, R.P.; Jamarun, N.; Rizqan, N.J.;. Production Performance, Feed Intake and Nutrient Digestibility of Etawa Crossbreed Dairy Goats Fed Tithonia (*Tithonia Diversifolia*), Cassava Leaves and Palm Kernel Cake Concentrate. *Int J Vet Sci* **2023**, *12*, 428–435, doi:10.47278/journal.ijvs/2022.211.
38. Arief, R.P.; Rizqan, N.J.; Magistri, P.M. Production Performance, Nutrient Digestibility and Food Consumption of Etawa Crosbreed Dairy Goats Fed *Gliricidia Sepium*, Concentrate of Palm Kernel Cake and Cassava Leaves. *Int J Vet Sci* **2024**, *13*, 471–478, doi:10.47278/journal.ijvs/2023.122.
39. Lakshmi, P.V.; Krishna, N. Evaluation of Complete Rations Containing Varying Levels of Palm Kernel-Cake as a Replacement for Groundnut-Cake in Sheep. *Indian J Anim Sci* **1995**, *65*, 1161–1164.
40. Umunna, N.N.; Magaji, I.Y.; Adu, I.F.; Njoku, P.C.; Balogun, T.F.; Alawa, J.P.; Iji, P.A. Utilization of Palm Kernel Meal by Sheep. *J Appl Anim Res* **1994**, *5*, 1–11, doi:10.1080/09712119.1994.9705991.
41. Carvalho, G.G.P.; Pires, A.J.V.; Silva, R.R.; Veloso, C.M.; Silva, H.G.O. Comportamento Ingestivo de Ovinos Alimentados com Dietas Compostas de Silagem de Capim-Elefante Amonizada ou Não e Subprodutos Agroindustriais. *Rev Bras Zootec* **2006**, *35*, 1805–1812, doi:10.1590/S1516-35982006000600031.
42. Costa, D.A.; Ferreira, G.D.G.; Araújo, C.V.; Colodo, J.C.N.; Moreira, G.R.; Figueiredo, M.R.P. Intake and Digestibility of Diets with Levels of Palm Kernel Cake in Sheep. *Rev Bras Saúde Prod Anim* **2010**, *11*, 783–792.
43. Bringel, L.M.L.; Neiva, J.N.M.; Araújo, V.L.; Bomfim, M.A.D.; Restle, J.; Ferreira, A.C.H.; Lôbo, R.N.B. Consumo, Digestibilidade e Balanço de Nitrogênio Em Borregos Alimentados com Torta de Dendê em Substituição à Silagem de Capim-Elefante. *Rev Bras Zootec* **2011**, *40*, 1975–1983, doi:10.1590/S1516-35982011000900019.

44. Macome, F.; Oliveira R.L; Regina, B.A.; Garcia-Leal, A.G.; Pires, B.L.; Costa-Alves, S.M. Productive Performance and Carcass Characteristics of Lambs Fed Diets Containing Different Levels of Palm Kernel Cake. *Rev MVZ Cordoba* **2011**, *16*, 2659–2667, doi:10.21897/rmvz.266.
45. Nunes, A.S.; Oliveira, R.L.; Borja, M.S.; Bagaldo, A.R.; Macome, F.M.; Jesus, I.B.; Silva, T.M.; Barbosa, L.P.; Garcez Neto, A.F. Consumo, Digestibilidade e Parâmetros Sanguíneos de Cordeiros Submetidos a Dietas com Torta de Dendê. *Arch Zootec* **2011**, *60*, 903–912, doi:10.4321/S0004-05922011000400007.
46. Macome, F.M.; Oliveira, R.L.; Araujo, G.G.L.; Barbosa, L.P.; Carvalho, G.G.P.; Garcez Neto, A.F.; Silva, T.M. Respostas de Ingestão e Fisiologicas de Cordeiros Alimentados com Torta de Dendê (Elaeis Guineensis). *Arch Zootec* **2012**, *61*, 335–342, doi:10.4321/S0004-05922012000300002.
47. Visoná-Oliveira, M.; Ferreira, I.C.; Macedo Junior, G.L.; Sousa, L.F.; Sousa, J.T.L.; Santos, R.P. Consumo e Digestibilidade de Nutrientes da Torta de Dendê na Dieta de Ovinos. *Cien Anim Bras* **2015**, *16*, 179–192, doi:10.1590/1089-6891V16I225615.
48. Pinho, B.D.; Ramos, A.F.O.; Lourenço Júnior, J.D.B.; Faturi, C.; Silva, A.G.M.; Nahúm, B.D.S.; Manno, M.C.; Lima, K.R.S.; Sousa, L.F.; Silva, J.A.R.; et al. Feeding Behavior of Sheep Fed Diets with Elaeis Guineensis Palm Kernel Meal. *Semin Cienc Agrar* **2016**, *37*, 2513, doi:10.5433/1679-0359.2016v37n4Supl1p2513.
49. Santos, R.C.; Alves, K.S.; Mezzomo, R.; Oliveira, L.R.S.; Cutrim, D.O.; Gomes, D.I.; Leite, G.P.; Araújo, M.Y.S. Performance of Feedlot Lambs Fed Palm Kernel Cake-Based Diets. *Trop Anim Health Prod* **2016**, *48*, 367–372, doi:10.1007/s11250-015-0960-y.
50. Omotoso, S.O.; Ajayi, F.T.; Kenneth-Obosi, O.; Oladele-Bukola, M.O. Nutritional Potential of Kenaf Grain Meal as a Replacement for Palm Kernel Cake in Cassava Peel-Based Concentrate for Sheep. *Agric Trop Subtrop* **2021**, *54*, 174–183, doi:10.2478/ats-2021-0018.
51. Castro, V.C.G.; Budel, J.C.C.; Rodrigues, T.C.G.C.; Silva, B.A.; de Lima, A.C.S.; de Souza, S.M.; Silva, J.A.R.; Joele, M.R.S.P.; Silva, A.G.M.; Lourenço-Junior, J.B. Nutrient Intake, Digestibility, Performance, Carcass Traits and Sensory Analysis of Meat from Lambs Fed with Co-Products of Amazon Oilseeds. *Front Vet Sci* **2023**, *10*, doi:10.3389/fvets.2023.1181765.
