# Supplementary material for: Effects of Palm Kernel Cake on Nutrient Utilization and Performance in Confined Cattle, Sheep and Goats: A Comparative Meta-Analytical Approach
Source: Animals (Basel). 2025 Sep 22;15(18):2764. doi: 10.3390/ani15182764 (PMC12466351; doi:10.3390/ani15182764)
Supplement: Supplementary file 1 [file animals-15-02764-s001.zip › Table S2.pdf]

Table S2. Mean values of nutritional components of experimental diets used in the studies included in the meta-analysis

Chemical composition (g/kg DM)	Mean	Standard deviation	Minimum	Maximum
Dry matter	733.3	19.7	249.1	962.0
Organic matter	853.6	10.3	519.8	962.7
Crude protein	143.0	2.99	54.4	220.7
Ether extract	37.63	2.09	7.50	165.5
Neutral detergent fiber	458.3	11.9	194.4	777.0
Acid detergent fiber	268.6	13.4	62.0	753.0
Ash	54.80	2.48	8.00	125.6
Non-fiber carbohydrates	343.2	11.2	93.3	608.6
