# Supplementary material for: Effects of Palm Kernel Cake on Nutrient Utilization and Performance in Confined Cattle, Sheep and Goats: A Comparative Meta-Analytical Approach
Source: Animals (Basel). 2025 Sep 22;15(18):2764. doi: 10.3390/ani15182764 (PMC12466351; doi:10.3390/ani15182764)
Supplement: Supplementary file 1 [file animals-15-02764-s001.zip › Table S3.pdf]

Table S3. Descriptive statistics of the chemical composition of palm kernel cake used in the studies with cattle, goats, and sheep

**Chemical composition (g/kg DM)**	**Mean**	**Standard deviation**	**Minimum**	**Maximum**
Dry matter	922.0	20.62	883.8	959.0
Crude protein	150.1	18.09	106.0	187.2
Ether extract	99.5	32.29	68.2	186.4
Neutral detergent fiber	655.8	76.08	548.1	818.5
Acid detergent fiber	428.7	75.88	219.6	560.2
Ash	49.8	30.11	21.0	146.0
Non-fiber carbohydrates	79.0	54.58	15.3	199.0
